# Discovery and Validation of Molecular Biomarkers for Differentiation of Nondysplastic Barrett’s Esophagus from High-grade Dysplasia and Esophageal Adenocarcinoma

**DOI:** 10.1158/1940-6207.CAPR-25-0215

**Published:** 2025-10-07

**Authors:** Caroline L. Matchett, Seth W. Slettedahl, William R. Taylor, Calise K. Berger, Caryn E. Anderson, Melissa A. Passe, Ramona M. Lansing, Panwen Wang, Collin E. Chalmers, Patrick H. Foote, Jeanette E. Eckel-Passow, Zhifu Sun, Douglas W. Mahoney, D. Chamil Codipilly, Cadman L. Leggett, Francisco C. Ramirez, Allon Kahn, Herbert C. Wolfsen, Swathi Eluri, Vani J.A. Konda, Arvind J. Trindade, Prasad G. Iyer, John B. Kisiel

**Affiliations:** 1 Division of Gastroenterology and Hepatology, Mayo Clinic, Rochester, Minnesota.; 2 Division of Clinical Trials and Biostatistics, Mayo Clinic, Rochester, Minnesota.; 3 Department of Quantitative Health Sciences, Mayo Clinic, Phoenix, Arizona.; 4 Division of Computational Biology, Mayo Clinic, Rochester, Minnesota.; 5 Division of Gastroenterology and Hepatology, Mayo Clinic, Scottsdale, Arizona.; 6 Division of Gastroenterology and Hepatology, Mayo Clinic, Jacksonville, Florida.; 7 Division of Gastroenterology and Hepatology, Baylor Scott and White Health, Dallas, Texas.; 8 Division of Gastroenterology, Rutgers University School of Medicine, New Brunswick, New Jersey.

## Abstract

Aberrant DNA methylation and copy-number alterations (CNA) drive Barrett’s esophagus progression to esophageal adenocarcinoma; however, their combined utility for early detection is unclear. We aimed to identify and validate methylated DNA markers (MDM) and CNAs to distinguish esophageal adenocarcinoma/high-grade dysplasia (HGD) from nondysplastic Barrett’s esophagus (NDBE). In this multiphase, multicenter study, we discovered and validated MDMs and quantified CNAs utilizing whole-genome methylation sequencing of esophageal brushings. DNA biomarkers identified from discovery were further validated in independent patients with paired esophageal brushing and swallowed capsule sponge samples. MDMs were filtered against a reduced representation bisulfite sequencing dataset obtained from independent tissue samples to advance only concordant candidates. CNA burden was quantified using ichorCNA-derived aneuploidy scores (AS). Two hundred MDMs discovered in HGD (*N* = 18) and esophageal adenocarcinoma (*N* = 18) versus NDBE brushing samples (*N* = 18) were tested in independent samples (*N* = 146). A 52-MDM panel achieved a cross-validated AUC of 0.88 [95% confidence interval (CI), 0.82–0.95]; the addition of AS improved discrimination of HGD/esophageal adenocarcinoma from NDBE to 0.91 (95% CI, 0.86–0.97) AUC. At 80% specificity, the combined model detected 93% of esophageal adenocarcinoma and 88% of HGD cases. In paired capsule sponge samples, a 58-MDM panel achieved a cross-validated AUC of 0.77 (95% CI, 0.66–0.88); a combined 58-MDM and AS model achieved AUC 0.80 (95% CI, 0.7–0.9). MDMs and AS discerned HGD/esophageal adenocarcinoma from normal esophagus/NDBE in endoscopic brushing and capsule sponge samples. This approach may improve Barrett’s esophagus surveillance.

**Prevention Relevance:**

This study demonstrates that combining epigenetic and genomic biomarkers across minimally invasive sampling methods can accurately distinguish HGD/esophageal adenocarcinoma from nondysplastic Barrett’s esophagus, offering promising, less invasive strategies to improve BE surveillance and enable endoscopic therapy for esophageal adenocarcinoma prevention and treatment.

## Introduction

Esophageal adenocarcinoma is a growing public health concern, with its incidence increasing dramatically in recent decades. In the United States, more than 20,000 individuals are diagnosed with esophageal cancer each year, and most of these cases are esophageal adenocarcinoma. The prognosis of esophageal adenocarcinoma diagnosed after the onset of symptoms is dismal, with 5-year survival rates below 20% (1–3). However, when detected at an early stage, outcomes improve dramatically; stage I esophageal adenocarcinoma treated with endoscopic or surgical intervention has 5-year survival rates of 80% to 90% (4). These stark contrasts in outcomes underscore the urgent need for more effective screening and early detection strategies.

Barrett's esophagus is the only established precursor to esophageal adenocarcinoma, progressing through a well-defined histologic sequence from nondysplastic Barrett's esophagus (NDBE) to low-grade dysplasia (LGD), high-grade dysplasia (HGD), and ultimately esophageal adenocarcinoma (5). Current prevention strategies rely on endoscopic surveillance with esophagogastroduodenoscopy (EGD) using the Seattle biopsy protocol (6) of patients with Barrett's esophagus to detect dysplasia or early-stage esophageal adenocarcinoma. Early identification enables timely intervention through endoscopic or surgical treatment aimed at halting the disease progression.

The current surveillance paradigm has some notable limitations. The Seattle protocol is subject to inherent sampling errors due to the heterogeneous distribution of dysplasia and esophageal adenocarcinoma within Barrett's esophagus mucosa (7). Although targeted biopsies from visibly abnormal areas are recommended, dysplasia can be endoscopically indistinct, and even esophageal adenocarcinoma may go undetected (8). Furthermore, there is significant interobserver variability in the histopathologic grading of dysplasia, which further complicates diagnosis and risk stratification (6, 9). Due to these limitations, HGD and early-stage esophageal adenocarcinoma are missed in up to 30% of cases, and the effectiveness of endoscopic surveillance in reducing esophageal adenocarcinoma mortality remains limited (10–13).

**Figure 1.**
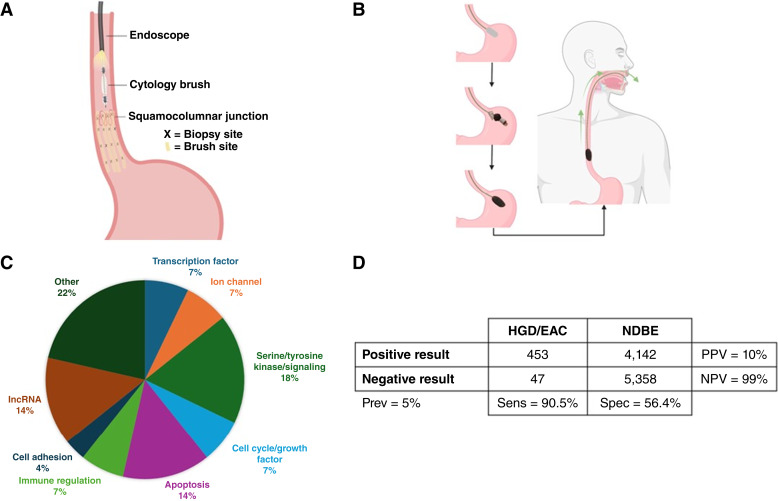
**A,** Illustrations of tissue sampling methods using (**A**) esophageal brushing (increasing surface area of sampled Barrett’s esophagus (BE) mucosa compared with Seattle protocol biopsies) and (**B**) a swallowable sponge device which samples the BE segment circumferentially in a nonoperator-dependent manner. **C,** Pie chart illustrating the functional categories of the 28 genes that overlapped in both brush and sponge validation experiments. **D,** Confusion matrix to estimate NPV and PPV in a hypothetical cohort of 10,000 patients with BE based on an assumed 5% prevalence of HGD/esophageal adenocarcinoma (EAC) and performance features of the 58-MDM + AS model on swallowed capsule sponge samples. lncRNA, long noncoding RNA; Prev, previous; Sens, sensitivity; Spec, specificity. [**A** and **B,** Created in BioRender. Matchett, C. (2025) https://BioRender.com/ y5a62oe]

To overcome the limitations of traditional biopsy-based surveillance, there is a critical need for complementary strategies that improve the detection of dysplasia and early-stage esophageal adenocarcinoma. Emerging advances in both sampling techniques and molecular biomarkers offer promising solutions. In particular, the use of molecular biomarkers measured from endoscopic brushings and swallowable esophageal cell collection devices that sample the esophageal mucosa has gained attention as potential solutions to enhance the sensitivity and consistency of Barrett’s esophagus sampling (Fig. 1; refs. 14, 15). These approaches provide a broader and more comprehensive sampling of the Barrett’s segment, addressing the patchy distribution of dysplasia that limits the effectiveness of the Seattle protocol. Molecular biomarkers such as methylated DNA markers (MDM) have emerged as highly sensitive indicators of neoplastic progression (16, 17). Methylation of specific CpG loci contributes to carcinogenesis by silencing tumor suppressor genes and promoting the transition from benign to malignant epithelium (18, 19).

Our previous work has demonstrated that MDMs can reliably distinguish Barrett’s esophagus—with or without dysplasia—and esophageal adenocarcinoma from normal squamous epithelium in biopsy specimens (20–23). Additionally, other research suggests that MDMs may complement other molecular alterations, such as somatic DNA copy-number alterations (CNA), to improve the discrimination of neoplastic Barrett’s esophagus from NDBE (15, 24). The understanding of how these molecular biomarkers perform in minimally invasive sampling contexts, such as endoscopic brushings and nonendoscopic capsule sponge devices, remains limited. Furthermore, the combined utility of MDMs and CNAs within these sampling platforms has not been fully characterized.

In the present study, our objectives were to

1. Discover novel MDMs and assess the association of CNA with HGD and esophageal adenocarcinoma using next-generation sequencing of DNA from endoscopic brush samples.
2. Validate the ability of these biomarkers to distinguish HGD/esophageal adenocarcinoma from NDBE and normal esophagus (NE) in an independent cohort of endoscopic brush samples.
3. Assess the performance of the validated biomarkers in paired samples collected using swallowable capsule sponge devices.
4. Evaluate whether combining MDMs and CNAs improves the accuracy of distinguishing HGD/esophageal adenocarcinoma from NDBE in these methods.

## Materials and Methods

### Overview

**Figure 2.**
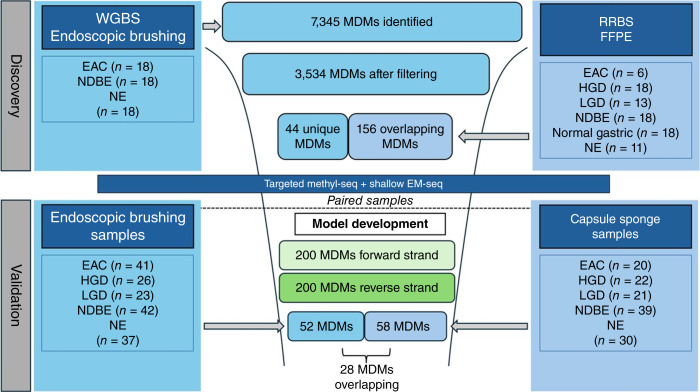
Multistage study design from marker discovery to validation. Workflow overview of initial marker discovery using WGMS on DNA extracted from endoscopic brushing samples with cross-reference to an archival RRBS dataset from DNA extracted from independent tissue samples, and independent validation comparing paired endoscopic brushings and sponge-on-string samples. EAC, esophageal adenocarcinoma; EM-seq, enzymatic methyl sequencing; methyl-seq, methyl sequencing; WGBS, whole genome bisulfite sequencing.

This study employed a sequential case–control design within a multi-institution project aimed at developing biomarkers to discriminate HGD and esophageal adenocarcinoma from NDBE (ClinicalTrials.gov identifiers: NCT02560623 and NCT03961945). The study proceeded in three phases. First, we conducted unbiased WGMS on DNA extracted from endoscopic esophageal brushings to identify candidate MDMs. These were filtered against a tissue-based reduced representation bisulfite sequencing (RRBS) dataset. Second, we validated the selected MDMs using an independent cohort of esophageal brushing samples. Third, we validated the selected MDMs in paired samples collected using a capsule sponge device from the same individuals included in the esophageal brushing validation cohort. In parallel, we performed CNA analysis with quantification by the ichorCNA (RRID: SCR_024768)-derived aneuploidy scores (AS) from whole-genome sequencing data generated in each phase of the study (Fig. 2).

The Mayo Clinic Institutional Review Board approved all study procedures (protocol #21-001489). This study was conducted and reported in accordance with the Standards for Reporting of Diagnostic Accuracy Studies (STARD) guidelines (Supplementary Table S1; ref. 25).

### Patients and samples

#### Discovery

Esophageal endoscopic brushing samples were collected from participants undergoing clinically indicated EGD at six United States medical institutions between January 2018 and April 2021. The institutions included Mayo Clinic (Minnesota, Arizona, and Florida), Mayo Clinic Health System Hospital (Austin, Minnesota), Northwell Health (New Hyde Park, New York), and Baylor University (Dallas, Texas). Samples were obtained from visible Barrett’s esophagus mucosa, with one brush used per 5-cm segment. Inclusion criteria for NDBE cases required participants to have ≥1 cm of circumferential BE and a history of at least one prior and one subsequent surveillance endoscopy without evidence of dysplasia to minimize the likelihood of missed prevalent dysplasia. For patients with HGD or esophageal adenocarcinoma, brush samples were collected from treatment-naive patients before endoscopy, surgery, radiation, or chemotherapy interventions. Healthy control samples were obtained from individuals without any history of esophageal neoplasia or Barrett’s esophagus from whom brushing samples were collected from the distal 5 cm of the squamous epithelium and cardia. All brushes were placed into a cell lysis buffer solution (Gentra Puregene Buccal Cell Kit, Qiagen) and stored at −80°C until further processing.

#### RRBS tissue

Formalin-fixed, paraffin-embedded (FFPE) tissue samples (18 esophageal adenocarcinoma cases, 18 HGD cases, 18 NDBE cases, and 18 normal controls) were obtained from the Mayo Clinic Tissue Registry and macrodissected prior to histologic review by an expert gastrointestinal pathologist.

#### Endoscopic brush validation

Additional endoscopic brushing samples were collected from independently recruited Barrett’s esophagus cases and healthy controls**.** The Barrett’s esophagus cases included participants with NDBE, LGD, HGD, and esophageal adenocarcinoma. For all participants, research biopsies were obtained after brushings and submitted for histopathologic review by an expert gastrointestinal pathologist to confirm the diagnosis and determine case or control status. In cases in which Barrett’s esophagus segments exceeded 5 cm, multiple brushes were used, and all DNA extracted from brushing samples from the same patient was pooled, mixed, and analyzed as a single sample, such that all samples had the same input DNA mass (see “Laboratory methods and assays,” “Discovery,” below).

#### Capsule sponge validation

Before EGD, all patients included in the endoscopic brush validation provided consent to undergo administration of a swallowable capsule sponge (EsophaCap, 25 mm diameter; CapNostics LLC), as previously described (20, 21). Sponge samples were collected before EGD with endoscopic brush sampling, typically on the same day within the same episode of care. After retrieval, the sponge was placed in a vial containing 20 mL of a cell preservative buffer (PreservCyt; Hologic) and stored at an ambient temperature until processing. Cells were pelleted and resuspended in the same lysis buffer used for the brushing samples and ultimately frozen at −80°C.

All participants included in this study provided signed written informed consent, and study procedures were carried out in accordance with the US Common Rule (45 CFR 46).

### Laboratory methods and assays

#### Discovery

DNA was extracted from endoscopic brush samples, and the Qiagen Puregene kit, modified to use 1 mL lysis buffer, was utilized. A measure of 120 ng of DNA per brush sample was sheared to 300 base pair (bp) fragments. Whole-genome methylation sequencing (WGMS) libraries were then prepared using the NEBNext Enzymatic Methyl-seq Kit (New England Biolabs). Converted libraries were amplified, quantified, and pooled in a 24-plex format. Three pooled libraries were sent to the Mayo Genome Analysis Core (RRID: SCR_024632) for sequencing on the Illumina NovaSeq system (Illumina) using S4 flow cells with paired-end sequencing reads of 150 bp in length. Results were analyzed for both CNA and methylation (below).

#### RRBS tissue

DNA was isolated from macrodissected tissue sections using the QIAamp FFPE Tissue Kit (Qiagen). DNA was purified using AMPure XP beads (Beckman Coulter) and quantified by PicoGreen (Thermo Fisher Scientific). DNA integrity was assessed using qPCR. RRBS libraries were prepared as described previously, with several modifications. For this study, we utilized the NuGEN Ovation RRBS Methyl-Seq kit (Tecan Genomics), which is applicable to degraded FFPE-derived DNA and obviates the need for a size selection step. After MspI digestion, adapter ligation, conversion (x2), and PCR enrichment, indexed samples were combined into 4-plex libraries and sequenced by the Mayo Genomics Facility on the Illumina HiSeq 4000 instrument (Illumina).

#### Endoscopic brush and capsule sponge validation

The validation dataset consisted of independently collected endoscopic brushings and matched capsule sponge samples from the same patients. Samples were centrifuged, and the resulting cell pellet was suspended in a lysis buffer. DNA from sponge lysates was extracted using the Maxwell RSC Blood DNA Kit (Promega) and brushings extracted as before. WGMS libraries for all samples were prepared as described above and divided into two fractions following enrichment. The first fraction (for CNA) underwent shallow WGMS on the Illumina NextSeq 2000 platform (RRID: SCR_023614) using P2 reagents. For the second fraction, selected regions (*n* = 200, described below) demonstrating differential methylation were enriched by target capture using a hybridization probe panel (IDT), prior to sequencing on the NextSeq 2000 platform (P1 reagents).

### Statistical and bioinformatic analysis

#### Discovery

Sample size requirements were based on the previously reported estimation method (26, 27). The WGMS data were preprocessed using Bismark (28) and in-house scripts. The raw sequencing reads were trimmed to remove potential sequencing adapter contamination and aligned to reference genome hg19 using bowtie. Methylation data for both CpG and non-CpG context cytosine were extracted, and quality control metrics (including alignment rate, number of captured CpGs, CpGs with ≥5× coverage, and bisulfite conversion rate) were generated. Only CpGs with at least 5× coverage were used in downstream analyses. Regions exhibiting dysplasia-associated differential CpG methylation (based on β-values) were identified using overdispersed logistic regression, with the NDBE cohort serving as a reference group. MDMs were ranked and filtered using the AUC, β-value case/control fold change [FC (%M case/%M control)], the absolute difference in Δ%M case–control, and statistical significance from the overdispersed logistic regression model. MDMs identified in the DNA extracted from endoscopic brush samples were cross-referenced with data from a previous RRBS experiment, performed on DNA extracted from FFPE tissue samples (Fig. 2).

Candidate regions shared by both datasets were considered further. The Integrative Genomics Viewer (Broad Institute, RRID: SCR_011793) was used to catalog MDMs with high concordance in CpG methylation across individual reads.

#### RRBS tissue

Reads were processed using Illumina pipeline modules for image analysis and base identification. Alignment, annotation, and methylation calling were conducted using SAAP-RRBS (RRID: SCR_006516), a Mayo-developed bioinformatics suite. Briefly, reads were cleaned-up using Trim Galore and aligned to the GRCh37/hg19 reference genome build with BSMAP. Methylation ratios were determined by calculating C/(C + T) or conversely, G/(G + A) for reads mapping to the reverse strand, for CpGs with coverage ≥10× and base quality score ≥20.

#### Endoscopic brush and capsule sponge validation

Bioinformatic preprocessing was performed as described above with the modification that a target bed file, limited to 200 MDMs, was used to assess enrichment efficiency (on-target read percentage) and average coverage across these regions. Sample size requirements were estimated based on methods outlined by McKeigue (29) and focused on classification using biomarker panels. The calculations considered the estimated proportion of MDMs with non-zero effects, the total number of MDMs investigated, the optimal predictive performance (based on AUC) of a model with infinite sample size, and the proportion of predictive information (based on the log-likelihood ratio) to be captured from the optimal model. Estimating an optimal AUC of 0.95, based on prior studies (20–22), and using 200 independent MDMs, we estimated that 20% of MDMs would have non-zero effects and intended to capture 55% of the predictive information (corresponding to an AUC of 0.89). Consequently, a minimum of 40 NDBE controls and 40 HGD/esophageal adenocarcinoma cases were required.

Methylation data from endoscopic brush and capsule sponge samples were analyzed independently. MDMs were filtered on variability observed in the NDBE control group from which the coefficient of determination was calculated for all MDMs using a linear model for markers with fewer than 30 CpGs and a generalized additive model for those with more than 30 CpGs. MDMs at or above the 80th percentile of the coefficient of determination were retained. For MDMs with both forward and reverse strand data (independently assessed) present after the filtering process, the strand with the larger coefficient of determination was retained to maintain independence across MDMs. Each filtered set of MDMs (brush and sponge considered independently) was then analyzed using random forest modeling. Sensitivity at 80% specificity and AUC were calculated using the predicted probability of HGD/esophageal adenocarcinoma case status. An 80% specificity threshold was chosen because of the high potential morbidity of missing HGD/esophageal adenocarcinoma and the low threshold for evaluating positive results with standard-of-care EGD.

Subsets of four MDMs, with and without AS (described below), were analyzed using logistic regression. This MDM limit was selected in anticipation of future conversion to higher-throughput targeted PCR-based assays, such as a long-probe quantitative amplified signal method that multiplexes classifier MDMs with a human DNA input normalizing marker (21). Optimism correction was performed on each 4-MDM combination, as detailed by Harrell and colleagues (30), and accuracy metrics were summarized. The effect of covariates [sex, age, body mass index (BMI), and smoking status] on model accuracy was assessed by comparing the differences in AUCs using a nonparametric test as described by DeLong and colleagues (31).

#### CNA detection

The following method was applied to both endoscopic brush discovery and endoscopic brush/capsule sponge validations. CNA detection was performed using the copy-number calling algorithm, ichorCNA (32). Uniquely mapped reads (with a mapping quality score >20) were extracted from the aligned BAM files, which were generated from the previously described methylation processing pipeline for both regular and shallow WGMS, using a bin size of 1 million base pairs (33, 34). A pooled reference was created from the NDBE control samples and used as the denominator to calculate a log_2_ ratio. ichorCNA uses HMMcopy to calculate, normalize, and perform GC correction (32). A hidden Markov model was used to segment the genome, detect large-scale CNAs (at least 5 Mb), and estimate the tumor fraction for each sample (esophageal adenocarcinoma, HGD, LGD, or NDBE, referent to normal squamous esophagus).

The AneuploidyScore (35, 36) R package (https://github.com/quevedor2/aneuploidy_score, RRID: SCR_001905) was used to calculate an AS for each sample to represent the total number of chromosome arms gained or lost in a sample (autosome chromosomes only), with a potential range from 0 to 44. The AS was calculated using the Cohen-Sharir method (36) in which the segment-size weighted median total copy number of each chromosome arm was assigned a loss/neutral/gain status (−1/0/1) based on its relation to the cell’s ploidy ± 0.5 threshold. The segment data were then reduced into the chromosome arm level as a singular value (−1, 0, or 1), and the AS was determined as the sum of the absolute value of arm data for a sample. In secondary analyses, six chromosome arm alterations, previously reported to be strongly associated with HGD/esophageal adenocarcinoma, were evaluated (1q gain, 9p loss, 12p gain, 17p loss, 20q gain, and 8q24 gain; ref. 15).

#### Complementarity of MDMs and CNA

To assess the complementarity of MDMs and CNA to predict group assignment, random forest models were generated using the set of MDMs and AS. Models were created and evaluated independently for endoscopic brush and capsule sponge samples using AUC and sensitivity at a set specificity of 80%.

#### Analysis of a rule-out test threshold

For capsule sponge data, a secondary analysis was performed to identify a test positive threshold that optimized sensitivity for HGD/esophageal adenocarcinoma. Model sensitivity was set at 90% for HGD/esophageal adenocarcinoma and estimated specificity to target a high negative predictive value (NPV) when applied to a future intended use population.

## Results

### Discovery

For the endoscopic brushing WGMS study, we generated 527 million mapped deduplicated reads/sample on average and 24 million CpGs per sample at 50× average depth of coverage.

### RRBS tissue sequencing

RRBS yielded ∼50 million mapped reads per sample on average, and 4 million CpGs per sample at 10× or greater coverage. After employing our marker discovery pipeline algorithm, logistic analysis, and performance (AUC) filtering, 4,501 MDMs were identified with an average length of 156 bp. These mapped to 1,549 annotated genes and 758 non-annotated regions of the genome. Further filtering to remove MDMs with excess methylation background noise brought the number of annotated and non-annotated regions to 1,196.

### Methylation

We identified 7,345 regions with differential methylation between cases (esophageal adenocarcinoma and HGD) and controls (NDBE and NE). The average length of these regions was approximately 72 bp. These potential MDMs mapped to 2,351 annotated genes and 1,374 non-annotated genomic regions. To refine the panel, we discarded regions with AUC < 0.60 and FC < 3×. In addition, regions with elevated stochastic methylation noise (<5%) in nondysplastic control samples were removed. This resulted in 3,534 candidate MDMs (Fig. 2). To drill down further, mitigate overfitting, and identify *bona fide* sites of cancer/dysplasia-associated hypermethylation, these 3,534 candidate MDMs were compared and merged with the RRBS set of 1,196 MDMs. To be considered further, the genomic loci of the MDMs from the two independent studies were required to be within 500 bases of each other (CpG islands can extend up to 1 Kb). Two hundred twenty-seven MDMs qualified, and the majority of them had overlapping loci. Using Integrative Genomics Viewer on BAM files from the alignment, the panel was narrowed further to 156 MDMs that exhibited ≥80% of CpGs methylated per strand at the sequencing read level for HGD and esophageal adenocarcinoma samples and ≤20% in the NDBE and NE cohorts. The remaining 3,307 WGMS brushing MDMs were ranked by AUC and FC metrics, and the top 44 were added to the panel for a final count of 200 MDMs. As RRBS is an enrichment method which only queries a subset of the genome, a whole-genome approach should logically identify additional candidates. MDM AUCs in this discovery phase ranged between 0.75 and 0.93 for the brushing samples and 0.75 and 0.88 for the tissues. More than 95% of the MDMs mapped to genomic CpG islands with the observed/expected CpG ratio >0.6. The majority of these were situated in the promoter/5′ untranslated region location of their respective genes.

### CNA

**Figure 3.**
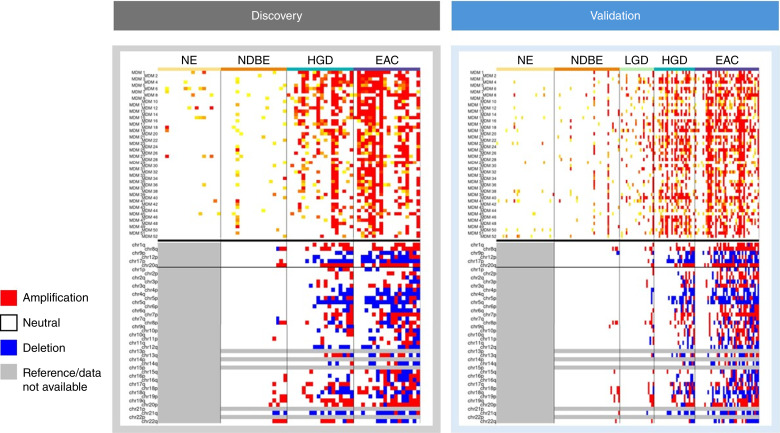
Methylation and copy-number profiles in discovery and validation case and control sample sets. For methylation, each row is a marker from the filtered set of 52 MDMs and each column a patient sample. For copy number, each row is a chromosomal arm. Methylation marker levels measured in deciles above the 95th percentile value in NDBE are denoted by the increasing intensity of yellow to red color spectrum. Copy-number analysis shows amplifications (red) or deletions (blue) referent to neutral (white) as estimated by the AS method. NDBE referent to NE (gray, neutral by definition). EAC, esophageal adenocarcinoma.

Referent to NE, which we assumed and observed to be euploid, chromosomal arm gains and losses were detected in 5 of 18 (28%) NDBE, 14 of 18 (78%) HGD, and 17 of 18 (94%) esophageal adenocarcinoma samples. The intensity and degree of chromosomal instability also generally increased with the level of dysplasia, with some HGDs and many esophageal adenocarcinomas exhibiting genome-wide alterations. Arm-level alterations in certain chromosomes were either recurrent gains or losses or a mix of both. We also looked specifically at six recurrent alterations that were identified by Douville and colleagues (15). These candidates (1q gain, 8q gain, 9p loss, 12p gain, 17p loss, and 20q gain) had been chosen because of previously reported cancer associations, with the 8q24 subregion gain being perhaps the most relevant to aneuploid esophageal adenocarcinomas. Our initial results replicated these observations, with the exception of 12p which tended toward many more losses than gains in our data (Fig. 3).

### Endoscopic brush validation

Independent endoscopic brush samples consisted of 41 esophageal adenocarcinoma, 26 HGD, 23 LGD, and 42 NDBE cases and 37 normal controls. One LGD sample was excluded because of inadequate quality of sequencing data. Patient clinical data are summarized in Table 1. For the target capture methylation libraries, the average sequencing depth of coverage for the 200-MDM genomic footprint was 336×. MDM performance (AUC, FC, and Δ%M case–control) and ranking generally correlated to the discovery numbers, though the increased coverage in the validation would be expected to confer improved granularity. To reduce the MDM panel to a statistically relevant number of markers for further analysis, given the number of samples in the validation set and the number of CpGs per marker, we calculated the coefficient of deviation for each MDM and filtered to the top 20%, figuring in both forward and reverse strands to account for strand-specific hemi-methylation and retaining only the strand with the higher deviation (see “Methods”), which yielded 52 MDMs (Fig. 3). For the CNA shallow whole-genome libraries, we sequenced to an average depth of coverage of 0.26×, almost 200-fold less than in the original discovery, but still more than sufficient for genome-wide aneuploidy detection. The resulting gain/loss trends were highly similar to those observed in the discovery phase. Esophageal adenocarcinomas again were the predominant cohort for the majority and intensity of CNA/aneuploidy disruptions, followed by HGDs and then LGDs (run here for the first time), with the NDBEs having the smallest proportion of observed events. The directionality distributions of the alterations (gains vs. losses or both) in the different cohorts were also maintained in the majority of chromosomal arms, including the six cancer-associated regions (Fig. 3).

**Table 1.** Baseline demographic characteristics of patients whose samples were included in the experiments.

Characteristic	Discovery				Validation				
	NE (*N* = 18)	NDBE (*N* = 18)	HGD (*N* = 18)	EAC (*N* = 18)	NE (*N* = 37)	NDBE (*N* = 42)	LGD (*N* = 23)	HGD (*N* = 26)	EAC (*N* = 41)
Age, mean (SD)	61 (12)	63 (8)	66 (8)	64 (10)	48 (13)	62 (11)	69 (10)	67 (8)	69 (11)
Race, *N* (%)	​	​	​	​	​	​	​	​	​
White	15 (83%)	18 (100%)	16 (89%)	18 (100%)	28 (76%)	38 (90%)	21 (91%)	24 (92%)	39 (95%)
Black	0 (0%)	0 (0%)	1 (5.5%)	0 (0%)	1 (22%)	0 (0%)	1 (4%)	0 (0%)	0 (0%)
Other	3 (17%)	0 (0%)	1 (5.5%)	0 (0%)	8 (3%)	4 (10%)	1 (4%)	2 (8%)	2 (5%)
Male sex, *N* (%)	11 (61%)	11 (61%)	16 (89%)	15 (83%)	22 (59%)	29 (69%)	17 (74%)	23 (88%)	35 (85%)
BMI kg/m^2^, mean (SD)	26 (4)	34 (7)	29 (7)	30 (6)	27 (7)	29 (6)	31 (7)	30 (5)	32 (6)
Ever-smoker, *N* (%)	9 (50%)	9 (50%)	14 (78%)	13 (72%)	16 (43%)	18 (43%)	17 (74%)	19 (73%)	29 (71%)
BE length (cm), mean (SD)​	—	5 (3)	6 (3)	5 (4)	—	4 (4)	5 (3)	4 (3)	5 (3)
Stage, *N* (%)	​	​	​	​	​	​	​	​	​
I	—	—	—	12 (67%)	—	—	—	—	29 (71%)
II	—	—	—	1 (5.5%)	—	—	—	—	4 (10%)
III	—	—	—	4 (22%)	—	—	—	—	6 (15%)
IV	—	—	—	1 (5.5%)	—	—	—	—	1 (2%)
Missing	—	—	—	—	—	—	—	—	1 (2%)
Sponge available, *N* (%)	—	—	—	—	30 (81%)	39 (93%)	21 (91%)	22 (85%)	20 (49%)

Abbreviations: BE, Barrett’s esophagus; EAC, esophageal adenocarcinoma.

**Figure 4.**
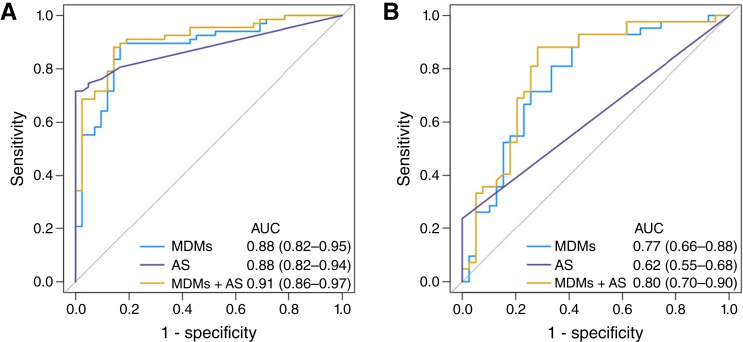
ROC curves for MDM performance in paired endoscopic brush and sponge samples. **A,** AUC analysis demonstrating discrimination of HGD/esophageal adenocarcinoma (EAC) from NDBE using the MDM panel, AS, and the combination in endoscopic brush samples. **B,** Corresponding AUC analysis showing marker performance in paired available (Table 1) sponge-on-string samples from the same patients.

A random forest model using a filtered list of 52 MDMs achieved a cross-validated AUC of 0.88 (0.82–0.95) for the identification of HGD/esophageal adenocarcinoma. AS generated an AUC of 0.88 (0.82–0.94) when comparing NDBE with HGD/esophageal adenocarcinoma. Combining the 52 MDMs and AS achieved a cross-validated AUC of 0.91 (0.86–0.97) for the discrimination of HGD/esophageal adenocarcinoma (Fig. 4A). At a specificity threshold of 80%, the 52-MDM model identified 38 esophageal adenocarcinomas [93% (79%–98%)], 22 HGDs [85% (64%–95%)], and 15 LGDs [68% (45%–85%)]. The 52-MDM and AS model had similar sensitivity, identifying 38 esophageal adenocarcinomas [93% (79%–98%)], 22 HGDs [85% (64%–95%)], and 16 LGDs [73% (50%–88%)].

### Capsule sponge validation

Patient-matched DNA from capsule sponge samples was available from 20 esophageal adenocarcinoma, 22 HGD, 21 LGD, and 39 NDBE cases and 30 normal controls. The sequencing approach was identical to that performed for the endoscopic brushing marker validation. For the targeted methylation panel, we achieved a 437× depth of coverage—moderately more than that with the prior samples. Other sequencing quality and performance metrics between the two validations were very similar. The sponge data, however, were less robust than the brushing data, most notably in MDM positivity. The majority of esophageal adenocarcinoma and HGD samples showed 2- to 10-fold lower MDM levels in sponges compared with brushes. As before, the data from the 200 MDMs were filtered by applying the coefficient of deviation analysis to the data, resulting here in a 58-MDM panel. For the shallow whole-genome libraries, the depth of coverage was 0.33×. As with the methylation results, the ichorCNA and AS readouts from the sponge samples were substantially inferior to the data from the brushes. Only six esophageal adenocarcinomas and four HGDs had observable aneuploidy, and all LGDs and NDBEs were negative. The results from the six CNA markers of interest correlated directionally with the earlier data but only in a subset of patients in which it was detected at all. In all cases in which CNA was observed, the intensity of the AS was diminished.

A random forest model with the filtered panel of 58 MDMs produced a cross-validated AUC of 0.77 (0.66–0.88) for discriminating HGD/esophageal adenocarcinoma from NDBE. AS produced an AUC of 0.62 (0.55–0.68). The combination of the 58-MDM panel and AS generated a cross-validated AUC of 0.80 (0.7–0.9; Fig. 4B). Using a set specificity of 80%, the 58-MDM model correctly classified 11 esophageal adenocarcinomas [55% (32%–76%)], 12 HGDs [55% (33%–75%)], and 12 LGDs [57% (34%–77%)]. The 58-MDM and AS model correctly identified 12 esophageal adenocarcinomas [60% (36%–80%)], 11 HGDs [50% (29%–71%)], and 13 LGDs [62% (39%–81%)], at a specificity cutoff of 80%.

### Rule-out test threshold analysis on capsule sponge data

Using a set sensitivity of 90% for HGD/esophageal adenocarcinoma, the MDM model achieved a specificity of 56% (40%–72%) for NDBE. Sensitivity for LGD was 86% (63%–96%). Using the same sensitivity cutoff of 90% for HGD/esophageal adenocarcinoma, the MDM and AS model produced similar specificity of 56% (40%–72%) in NDBE and a slightly lower sensitivity for LGD, 81% (57%–94%).

### Reducing the marker panel

Of the 52 MDMs in the endoscopic brush validation panel and the 58 MDMs in the capsule sponge validation panel, 28 MDMs were shared between both models. Using the 28 shared MDMs, all possible combinations of four markers were evaluated to assess the classification performance of a reduced 4-marker panel, based on data from the endoscopic brush validation cohort. The median optimism-corrected AUC from more than 20,000 4-MDM logistic regression models was 0.86 (IQR = 0.84–0.88). The median optimism-corrected sensitivity for HGD/esophageal adenocarcinoma was 80% (77%–83%). AS was added to all 20,000+ 4-MDM models, the median optimism-corrected AUC increased to 0.91 (0.90–0.92), and the median sensitivity increased to 88% (86%–89%). The molecular/biological function of the genes represented by the 28 MDMs was generally tumorigenic in character, including transcription factors, ion channels, apoptotic proteins, tyrosine kinases, and other signaling enzymes. Four were long noncoding RNAs. Most had prior cancer associations in the literature, mainly via altered gene expression observations and mutated sequences. As before, these MDMs mapped predominantly to promoter/5′ untranslated region regulatory sequences (Fig. 1).

### Analysis of covariates

Stratified AUCs were calculated for the 52-MDM model, stratifying by age, sex, BMI, and smoking status (Table 2). No statistically significant differences in AUC were observed when comparing participants >65 versus ≤65 years of age, male versus female, BMI >30 versus ≤30, or ever-smokers vs. never-smokers. The length of Barrett’s esophagus segment (short 1–2 cm vs. long 3 cm) was also not statistically significantly different using this method.

**Table 2.** AUC of the 52-MDM model in cytology brush validation data comparing NDBE vs. HGD/EAC, stratified by covariates.

Variable ​	Yes	No	*P* value
Age > 65 years	0.90 (0.74–0.96)	0.85 (0.80–0.99)	0.550
Male sex	0.88 (0.80–0.96)	0.88 (0.70–1.0)	0.972
BMI > 30 kg/m^2^	0.94 (0.88–1.0)	0.83 (0.71–0.94)	0.087
Ever-smoker	0.92 (0.85–0.99)	0.83 (0.70–0.96)	0.220
BE length ≥ 3 cm	0.91 (0.83–0.98)	0.87 (0.77–0.97)	0.561

Abbreviation: EAC, esophageal adenocarcinoma.

## Discussion

The increasing incidence of esophageal adenocarcinoma and substantial proportion of missed HGD and esophageal adenocarcinoma with current endoscopic biopsy-based strategies (Seattle protocol) highlight the pressing need to improve performance of Barrett’s esophagus surveillance methods (8, 12). Our study addresses these challenges through three key advances: (1) discovery and validation of novel MDMs that are discriminant for HGD and esophageal adenocarcinoma from NDBE, (2) demonstration that CNAs offer complementary diagnostic value, and (3) successful translation of these molecular approaches to both endoscopic brush and minimally invasive swallowable capsule sponge sampling methods.

Our analysis showed that combining MDMs with CNAs numerically enhanced the ability to distinguish HGD/esophageal adenocarcinoma from NDBE (AUC 0.91 vs. 0.88 with MDMs alone). The CNA analysis focused on recurrent alterations known to be associated with Barrett’s esophagus neoplasia, including gains in 1q, 12p, and 20q and losses in 9p and 17p—five of the six key regions previously identified by Douville and colleagues (15). The concordance between our findings and these established patterns supports the validity of our approach.

Additionally, we performed an *in silico* analysis to evaluate the performance of reduced 4-MDM models, simulating configurations compatible with a multiplexed PCR-based assay (e.g., TELQAS, Exact Sciences). Across more than 20,000 logistic regression models, the median optimism-corrected AUC was 0.86 (IQR = 0.84–0.88), with a corresponding median sensitivity for HGD/esophageal adenocarcinoma of 80% (IQR = 77%–83%). When AS was included alongside the 4 MDMs, the median optimism-corrected AUC improved to 0.91 (IQR = 0.90–0.92) and sensitivity increased to 88% (IQR = 86%–89%). These findings support further investigation of the potential complementary value of combining methylation and CNAs and demonstrate the feasibility of developing a streamlined, high-performance classifier suitable for clinical translation. Demonstrating this classification accuracy in endoscopic brushes is clinically meaningful because of the high proportion of HGD/esophageal adenocarcinoma missed at index surveillance EGD by targeted visual inspection and random biopsy (8), suggesting a need for wider mucosal sampling by brushing which may be additive to the current standard of care (Seattle biopsy protocol) alone.

A major innovation of our study was validating biomarker performance using a minimally invasive swallowed capsule sponge device. This approach offers several advantages over traditional endoscopic surveillance. It is less costly, better tolerated by patients, does not require sedation, enables circumferential mucosal sampling, and is scalable for use in primary care—particularly in settings where access to endoscopy is limited (37–40). However, unlike targeted endoscopic brushings, the capsule sponge samples a broader anatomic region, including the gastric cardia, esophageal squamous epithelium, and oropharynx, introducing greater cellular heterogeneity. To estimate the impact of this effect, we analyzed paired sponge and brush samples from the same individuals. This design allowed for a direct comparison of sampling methods while minimizing interpatient variability. The modest reduction in diagnostic accuracy observed in sponge samples (AUC = 0.77 for MDMs alone; 0.80 with MDMs + CNA) is likely attributable to this increased heterogeneity, but the results nonetheless support the feasibility of capsule sponge–based molecular testing for Barrett’s esophagus–related neoplasia. We performed a secondary analysis to assess the potential of our MDM and CNA models as a high-sensitivity rule-out test for Barrett’s esophagus–associated neoplasia in patients with limited access to endoscopic surveillance and prioritize endoscopic assessments for those with positive results. A similar approach has been investigated in the United Kingdom using the Cytosponge device, in which the absence of p53 overexpression and cytologic atypia—combined with demographic risk factors—was associated with a 99% NPV for HGD/esophageal adenocarcinoma using p53 (41). Notably, the ongoing BEST4 trial in the United Kingdom is now evaluating the real-world effectiveness of this capsule sponge device combined with biomarker testing for both screening and surveillance of Barrett’s esophagus/esophageal adenocarcinoma at a national scale (42). The high observed positive predictive value (PPV) of p53 and atypia in high-risk patients (31%) was directly related to the high prevalence of HGD/esophageal adenocarcinoma in that study’s test group (17%) which is >3-fold higher than that observed in southeastern Minnesota. If applying test thresholds from our model to an intended use Barrett’s esophagus population with 5% prevalence of HGD/esophageal adenocarcinoma, as we have previously measured in 11 southeastern Minnesota counties (43), the estimated NPV and PPV would be 99% and 11%, respectively (Fig. 1). This reinforces the potential for such a test to be used for selecting patients with Barrett’s esophagus most likely to harbor prevalent dysplasia/esophageal adenocarcinoma.

Several important considerations remain for future development and clinical translation. Although our initial marker panel demonstrates diagnostic promise, further refinement is needed to optimize clinical utility. Ongoing work will focus on *elimination* of redundant or low-contribution markers through validation in larger, independent cohorts—including NE, NDBE, and HGD/esophageal adenocarcinoma cases—and selection of the minimum number of informative markers necessary for robust algorithm performance. This step is particularly crucial for capsule sponge–based sampling, in which low neoplastic cell fraction may reduce detectable signal. Although our study used targeted next-generation sequencing for discovery and validation, translation to more scalable and cost-effective platforms—such as multiplexed qPCR—will require technical adaptation, algorithm development, analytic validation, and comparative cost-effectiveness analyses and prospective studies designed to assess predictive value in real-world clinical populations. Finally, although our findings suggest that combining CNA with MDMs enhances diagnostic performance, this study was not powered to verify the independent or additive contribution of CNA. Determining the optimal integration of these molecular features will be a key focus of future research.

In conclusion, we demonstrate the feasibility and diagnostic utility of combining methylation and genomic biomarkers to detect Barrett’s esophagus–associated neoplasia using both endoscopic and minimally invasive sampling methods. These results represent a meaningful advance toward more accurate, accessible, and scalable surveillance strategies for patients with Barrett’s esophagus.

## Supplementary Material

Supplemental Table 1 Supplemental Table 1: Study adherence to the STARD (Standards for Reporting Diagnostic Accuracy Studies) 2015 checklist.

## Data Availability

The data generated in this study are not publicly available because of prohibitions by our sponsored research agreement with Exact Sciences. Access to molecular level data from DNA sequencing or deidentified clinical data requires permission from Exact Sciences Corporation (Madison, WI; contact: Jorge Garces, jgarces@exactsciences.com), Mayo Clinic Legal Contract Administration (contact Randall Jones, jones.randall@mayo.edu), and the Mayo Clinic Institutional Review Board.
