# Supplementary material for: Discovery and Validation of Molecular Biomarkers for Differentiation of Nondysplastic Barrett’s Esophagus from High-grade Dysplasia and Esophageal Adenocarcinoma
Source: Cancer Prev Res (Phila). 2025 Oct 7;19(1):49–60. doi: 10.1158/1940-6207.CAPR-25-0215 (PMC12770938; doi:10.1158/1940-6207.CAPR-25-0215)
Supplement: Supplemental Table 1 — Study adherence to the STARD (Standards for Reporting Diagnostic Accuracy Studies) 2015 checklist. [file capr-25-0215_supplemental_table_1_suppst1.docx]

**Supplemental Table 1.** Study adherence to the STARD (Standards for Reporting Diagnostic Accuracy Studies) 2015 checklist.

| **Section & Topic** | **No** | **Item** | **Reported on page #** |
| --- | --- | --- | --- |
|  |  |  |  |
| **TITLE OR ABSTRACT** |  |  |  |
|  | **1** | Identification as a study of diagnostic accuracy using at least one measure of accuracy  (such as sensitivity, specificity, predictive values, or AUC) | 3 |
| **ABSTRACT** |  |  |  |
|  | **2** | Structured summary of study design, methods, results, and conclusions  (for specific guidance, see STARD for Abstracts) | 3 |
| **INTRODUCTION** |  |  |  |
|  | **3** | Scientific and clinical background, including the intended use and clinical role of the index test | 5 and 6 |
|  | **4** | Study objectives and hypotheses | 7 |
| **METHODS** |  |  |  |
| *Study design* | **5** | Whether data collection was planned before the index test and reference standard  were performed (prospective study) or after (retrospective study) | 8 |
| *Participants* | **6** | Eligibility criteria | 8 and 9 |
|  | **7** | On what basis potentially eligible participants were identified  (such as symptoms, results from previous tests, inclusion in registry) | 8 and 9 |
|  | **8** | Where and when potentially eligible participants were identified (setting, location and dates) | 8 and 9 |
|  | **9** | Whether participants formed a consecutive, random or convenience series | 8 and 9 |
| *Test methods* | **10a** | Index test, in sufficient detail to allow replication | 9-14 |
|  | **10b** | Reference standard, in sufficient detail to allow replication | 9-14 |
|  | **11** | Rationale for choosing the reference standard (if alternatives exist) | NA |
|  | **12a** | Definition of and rationale for test positivity cut-offs or result categories  of the index test, distinguishing pre-specified from exploratory | 9-14 |
|  | **12b** | Definition of and rationale for test positivity cut-offs or result categories  of the reference standard, distinguishing pre-specified from exploratory | 12-14 |
|  | **13a** | Whether clinical information and reference standard results were available  to the performers/readers of the index test | 9-14 |
|  | **13b** | Whether clinical information and index test results were available  to the assessors of the reference standard | 9-14 |
| *Analysis* | **14** | Methods for estimating or comparing measures of diagnostic accuracy | 11-14 |
|  | **15** | How indeterminate index test or reference standard results were handled | 11-14 |
|  | **16** | How missing data on the index test and reference standard were handled | 11-14 |
|  | **17** | Any analyses of variability in diagnostic accuracy, distinguishing pre-specified from exploratory | 11-14 |
|  | **18** | Intended sample size and how it was determined | 18 |
| **RESULTS** |  |  |  |
| *Participants* | **19** | Flow of participants, using a diagram | 8 and Figure 2 |
|  | **20** | Baseline demographic and clinical characteristics of participants | Table 1 |
|  | **21a** | Distribution of severity of disease in those with the target condition | 15 and 16 |
|  | **21b** | Distribution of alternative diagnoses in those without the target condition | 15 and 16 |
|  | **22** | Time interval and any clinical interventions between index test and reference standard | NA |
| *Test results* | **23** | Cross tabulation of the index test results (or their distribution)  by the results of the reference standard | 15-18 |
|  | **24** | Estimates of diagnostic accuracy and their precision (such as 95% confidence intervals) | 15-18 |
|  | **25** | Any adverse events from performing the index test or the reference standard | NA |
| **DISCUSSION** |  |  |  |
|  | **26** | Study limitations, including sources of potential bias, statistical uncertainty, and generalisability | 21 |
|  | **27** | Implications for practice, including the intended use and clinical role of the index test | 19-22 |
| **OTHER INFORMATION** |  |  |  |
|  | **28** | Registration number and name of registry | NA |
|  | **29** | Where the full study protocol can be accessed | 2 |
|  | **30** | Sources of funding and other support; role of funders | 2 |
|  |  |  |  |
